# 1-Hydr­oxy-2-meth­oxy-6-methyl-9,10-anthraquinone from *Rennellia elliptica* Korth.

**DOI:** 10.1107/S1600536809017619

**Published:** 2009-05-29

**Authors:** Nor Hadiani Ismail, Che Puteh Osman, Rohaya Ahmad, Khalijah Awang, Seik Weng Ng

**Affiliations:** a Faculty of Applied Sciences, Universiti Teknologi MARA, 40450 Shah Alam, Selangor Darul Ehsan, Malaysia; b Department of Chemistry, University of Malaya, 50603 Kuala Lumpur, Malaysia

## Abstract

The title compound, C_16_H_12_O_4_, exists as planar molecules in the solid state (r.m.s. deviation of 0.02 Å in one mol­ecule and 0.07 Å in the second independent mol­ecule comprising the asymmetric unit). In each mol­ecule, the 1-hydr­oxy group forms an intra­molecular hydrogen bond to the adjacent carbonyl O atom.

## Related literature

The existence of the title natural product has only been reported for *Crucianella maritima* L. (El-Lakany *et al.*, 2004 ▶). For another anthraquinone isolated from *Rennellia elliptica* Korth., see: Ismail *et al.* (2009 ▶).
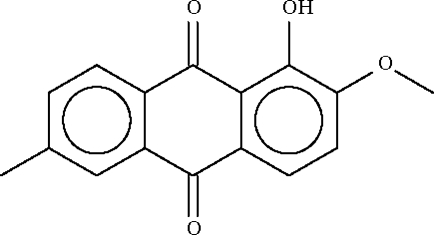


## Experimental

### 

#### Crystal data

C_16_H_12_O_4_
*M* *_r_* = 268.26
Triclinic, 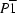

*a* = 7.1755 (3) Å
*b* = 11.9082 (5) Å
*c* = 14.9683 (7) Å
α = 91.409 (3)°
β = 100.603 (3)°
γ = 105.666 (3)°
*V* = 1206.73 (9) Å^3^
*Z* = 4
Mo *K*α radiation
μ = 0.11 mm^−1^
*T* = 100 K
0.25 × 0.20 × 0.01 mm

#### Data collection

Bruker SMART APEX diffractometer
Absorption correction: none
6750 measured reflections
4142 independent reflections
2248 reflections with *I* > 2σ(*I*)
*R*_int_ = 0.051

#### Refinement

*R*[*F* ^2^ > 2σ(*F* ^2^)] = 0.082
*wR*(*F* ^2^) = 0.267
*S* = 1.08
4142 reflections
367 parameters
H-atom parameters constrained
Δρ_max_ = 0.59 e Å^−3^
Δρ_min_ = −0.35 e Å^−3^

### 

Data collection: *APEX2* (Bruker, 2008 ▶); cell refinement: *SAINT* (Bruker, 2008 ▶); data reduction: *SAINT*; program(s) used to solve structure: *SHELXS97* (Sheldrick, 2008 ▶); program(s) used to refine structure: *SHELXL97* (Sheldrick, 2008 ▶); molecular graphics: *X-SEED* (Barbour, 2001 ▶); software used to prepare material for publication: *publCIF* (Westrip, 2009 ▶).

## Supplementary Material

Crystal structure: contains datablocks global, I. DOI: 10.1107/S1600536809017619/tk2448sup1.cif

Structure factors: contains datablocks I. DOI: 10.1107/S1600536809017619/tk2448Isup2.hkl

Additional supplementary materials:  crystallographic information; 3D view; checkCIF report

## Figures and Tables

**Table 1:** Hydrogen-bond geometry (Å, °)

*D*—H⋯*A*	*D*—H	H⋯*A*	*D*⋯*A*	*D*—H⋯*A*
O3—H3⋯O2	0.84	1.80	2.538 (4)	147
O7—H7⋯O6	0.84	1.81	2.551 (5)	146

**Fig. 1.**
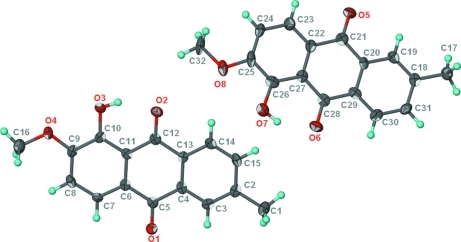
Thermal ellipsoid plot (Barbour, 2001) of the two independent molecules of C16H12O4 at the 70% probability level. Hydrogen atoms are drawn as spheres of arbitrary radius.

## supplementary crystallographic information

### Experimental

About 1 kg of the root of *Rennelia elliptica* Korth., which was collected from the Kuala Keniam National Park, Malaysia, was extracted with dichloromethane. The solvent was removed to give a crude material (approx. 10 g) that was fractionated on a chromatography column (60 x 5 cm) packed with silica. The silica had been previously immersed in 4% oxalic acid and then activated by heating to 363 K. The fractions were eluted with hexane–dichloromethane (3:7 v/v), and those fractions having an identical TLC pattern were combined and then subjected to column chromatography (330 x 15 mm), with dichloromethane as eluent. The compound was further purified on a short glass column (50 x 5 mm). The solvent was removed and the product recrystallized from chloroform to furnish yellow crystals (about 10 mg). The formulation was established by ^1^H- and ^13^C-NMR spectroscopy.

### Refinement

Hydrogen atoms were placed at calculated positions (C–H 0.95–0.98 Å) and were treated as riding on their parent carbon atoms, with *U*(H) set to 1.2–1.5 times *U*_eq_(*C*). The hydroxy H-atoms were similarly generated (O–H 0.84 Å).

### Crystal data

**Table:**

C_16_H_12_O_4_	*Z* = 4
*M**_r_* = 268.26	*F*(000) = 560
Triclinic, *P*1	*D*_x_ = 1.477 Mg m^−^^3^
Hall symbol: -P 1	Mo *K*α radiation, λ = 0.71073 Å
*a* = 7.1755 (3) Å	Cell parameters from 1159 reflections
*b* = 11.9082 (5) Å	θ = 2.8–26.8°
*c* = 14.9683 (7) Å	µ = 0.11 mm^−^^1^
α = 91.409 (3)°	*T* = 100 K
β = 100.603 (3)°	Plate, yellow
γ = 105.666 (3)°	0.25 × 0.20 × 0.01 mm
*V* = 1206.73 (9) Å^3^	

### Data collection

**Table:**

Bruker SMART APEX diffractometer	2248 reflections with *I* > 2σ(*I*)
Radiation source: fine-focus sealed tube	*R*_int_ = 0.051
graphite	θ_max_ = 25.0°, θ_min_ = 1.8°
ω scans	*h* = −8→8
6750 measured reflections	*k* = −14→13
4142 independent reflections	*l* = −17→17

### Refinement

**Table:**

Refinement on *F*^2^	Primary atom site location: structure-invariant direct methods
Least-squares matrix: full	Secondary atom site location: difference Fourier map
*R*[*F*^2^ > 2σ(*F*^2^)] = 0.082	Hydrogen site location: inferred from neighbouring sites
*wR*(*F*^2^) = 0.267	H-atom parameters constrained
*S* = 1.08	*w* = 1/[σ^2^(*F*_o_^2^) + (0.1397*P*)^2^ + 0.3435*P*] where *P* = (*F*_o_^2^ + 2*F*_c_^2^)/3
4142 reflections	(Δ/σ)_max_ = 0.001
367 parameters	Δρ_max_ = 0.59 e Å^−^^3^
0 restraints	Δρ_min_ = −0.35 e Å^−^^3^

### Fractional atomic coordinates and isotropic or equivalent isotropic displacement parameters (Å^2^)

**Table:**

	*x*	*y*	*z*	*U*_iso_*/*U*_eq_	
O1	0.2331 (5)	0.2601 (3)	0.5314 (2)	0.0207 (8)	
O2	0.2924 (5)	0.7172 (3)	0.4917 (2)	0.0219 (9)	
O3	0.3202 (5)	0.6932 (3)	0.3261 (2)	0.0205 (8)	
H3	0.3084	0.7272	0.3737	0.031*	
O4	0.3344 (5)	0.5601 (3)	0.1875 (2)	0.0207 (8)	
O5	0.9299 (6)	1.2275 (3)	1.0962 (2)	0.0327 (10)	
O6	0.5739 (6)	0.7665 (3)	1.0100 (2)	0.0281 (9)	
O7	0.5636 (6)	0.7944 (3)	0.8409 (2)	0.0297 (10)	
H7	0.5365	0.7587	0.8866	0.045*	
O8	0.6524 (5)	0.9285 (3)	0.7122 (2)	0.0279 (9)	
C1	0.1303 (8)	0.4169 (5)	0.8389 (3)	0.0247 (13)	
H1A	0.2063	0.3608	0.8555	0.037*	
H1B	−0.0110	0.3773	0.8316	0.037*	
H1C	0.1682	0.4806	0.8872	0.037*	
C2	0.1725 (7)	0.4662 (4)	0.7505 (3)	0.0188 (11)	
C3	0.1867 (7)	0.3958 (4)	0.6786 (3)	0.0186 (11)	
H3A	0.1746	0.3153	0.6858	0.022*	
C4	0.2183 (7)	0.4406 (4)	0.5959 (3)	0.0163 (11)	
C5	0.2389 (7)	0.3634 (4)	0.5212 (3)	0.0158 (11)	
C6	0.2613 (7)	0.4126 (4)	0.4328 (3)	0.0155 (11)	
C7	0.2703 (7)	0.3443 (4)	0.3595 (3)	0.0146 (11)	
H7A	0.2615	0.2640	0.3662	0.017*	
C8	0.2921 (7)	0.3884 (4)	0.2752 (3)	0.0187 (12)	
H8	0.2951	0.3385	0.2253	0.022*	
C9	0.3092 (7)	0.5067 (4)	0.2654 (3)	0.0179 (11)	
C10	0.3034 (7)	0.5792 (4)	0.3390 (3)	0.0160 (11)	
C11	0.2798 (7)	0.5340 (4)	0.4234 (3)	0.0153 (11)	
C12	0.2727 (7)	0.6111 (4)	0.4991 (3)	0.0176 (11)	
C13	0.2400 (7)	0.5601 (4)	0.5865 (3)	0.0143 (11)	
C14	0.2299 (7)	0.6314 (4)	0.6590 (3)	0.0184 (11)	
H14	0.2462	0.7125	0.6527	0.022*	
C15	0.1964 (7)	0.5858 (4)	0.7401 (3)	0.0195 (12)	
H15	0.1894	0.6355	0.7893	0.023*	
C16	0.3257 (8)	0.4888 (5)	0.1080 (4)	0.0295 (14)	
H16A	0.3329	0.5367	0.0558	0.044*	
H16B	0.2014	0.4261	0.0959	0.044*	
H16C	0.4370	0.4546	0.1179	0.044*	
C17	0.8428 (9)	1.0596 (5)	1.4057 (3)	0.0262 (13)	
H17A	0.9719	1.1182	1.4193	0.039*	
H17B	0.8450	0.9950	1.4449	0.039*	
H17C	0.7409	1.0957	1.4171	0.039*	
C18	0.7980 (7)	1.0137 (5)	1.3075 (3)	0.0204 (12)	
C19	0.8440 (7)	1.0856 (5)	1.2383 (3)	0.0204 (12)	
H19	0.9084	1.1664	1.2537	0.024*	
C20	0.7990 (7)	1.0432 (4)	1.1479 (3)	0.0167 (11)	
C21	0.8479 (8)	1.1216 (5)	1.0761 (3)	0.0221 (12)	
C22	0.7990 (7)	1.0732 (4)	0.9807 (3)	0.0206 (12)	
C23	0.8403 (8)	1.1438 (5)	0.9115 (3)	0.0230 (12)	
H23	0.9019	1.2251	0.9259	0.028*	
C24	0.7944 (8)	1.0992 (5)	0.8208 (3)	0.0236 (12)	
H24	0.8261	1.1500	0.7745	0.028*	
C25	0.7032 (8)	0.9821 (5)	0.7979 (3)	0.0236 (13)	
C26	0.6581 (7)	0.9084 (4)	0.8672 (4)	0.0218 (12)	
C27	0.7028 (7)	0.9509 (4)	0.9582 (3)	0.0193 (12)	
C28	0.6543 (7)	0.8737 (4)	1.0285 (3)	0.0196 (12)	
C29	0.7021 (7)	0.9217 (4)	1.1244 (3)	0.0196 (12)	
C30	0.6537 (7)	0.8511 (5)	1.1930 (3)	0.0221 (12)	
H30	0.5869	0.7705	1.1781	0.027*	
C31	0.7009 (8)	0.8955 (4)	1.2837 (3)	0.0224 (12)	
H31	0.6666	0.8449	1.3299	0.027*	
C32	0.6916 (9)	0.9996 (6)	0.6386 (4)	0.0317 (14)	
H32A	0.6461	0.9508	0.5809	0.047*	
H32B	0.8338	1.0370	0.6471	0.047*	
H32C	0.6216	1.0600	0.6374	0.047*	

### Atomic displacement parameters (Å^2^)

**Table:**

	*U*^11^	*U*^22^	*U*^33^	*U*^12^	*U*^13^	*U*^23^
O1	0.025 (2)	0.0202 (19)	0.0184 (19)	0.0103 (16)	0.0018 (15)	0.0029 (15)
O2	0.027 (2)	0.0162 (19)	0.023 (2)	0.0089 (16)	0.0025 (16)	0.0009 (15)
O3	0.026 (2)	0.0153 (18)	0.020 (2)	0.0053 (16)	0.0048 (16)	0.0001 (15)
O4	0.023 (2)	0.025 (2)	0.0140 (19)	0.0080 (16)	0.0030 (15)	−0.0005 (15)
O5	0.048 (3)	0.018 (2)	0.025 (2)	−0.0013 (19)	0.0036 (19)	−0.0023 (16)
O6	0.035 (2)	0.018 (2)	0.028 (2)	0.0044 (18)	0.0015 (17)	−0.0022 (16)
O7	0.037 (2)	0.026 (2)	0.024 (2)	0.0076 (19)	0.0049 (18)	−0.0012 (17)
O8	0.033 (2)	0.038 (2)	0.014 (2)	0.0150 (19)	−0.0003 (16)	−0.0025 (17)
C1	0.021 (3)	0.033 (3)	0.024 (3)	0.010 (3)	0.011 (2)	0.007 (2)
C2	0.011 (3)	0.028 (3)	0.017 (3)	0.007 (2)	−0.001 (2)	0.003 (2)
C3	0.012 (3)	0.020 (3)	0.023 (3)	0.005 (2)	0.000 (2)	0.005 (2)
C4	0.006 (2)	0.025 (3)	0.017 (3)	0.005 (2)	−0.002 (2)	−0.004 (2)
C5	0.008 (3)	0.020 (3)	0.019 (3)	0.005 (2)	0.000 (2)	0.000 (2)
C6	0.008 (3)	0.022 (3)	0.015 (3)	0.005 (2)	−0.003 (2)	0.000 (2)
C7	0.010 (3)	0.013 (2)	0.020 (3)	0.002 (2)	0.004 (2)	0.001 (2)
C8	0.016 (3)	0.023 (3)	0.016 (3)	0.008 (2)	−0.001 (2)	−0.005 (2)
C9	0.013 (3)	0.025 (3)	0.016 (3)	0.005 (2)	0.002 (2)	0.004 (2)
C10	0.010 (3)	0.022 (3)	0.015 (3)	0.004 (2)	−0.001 (2)	0.004 (2)
C11	0.011 (3)	0.018 (3)	0.015 (3)	0.007 (2)	−0.004 (2)	−0.001 (2)
C12	0.010 (3)	0.022 (3)	0.019 (3)	0.004 (2)	−0.002 (2)	0.000 (2)
C13	0.007 (2)	0.021 (3)	0.016 (3)	0.007 (2)	0.0013 (19)	0.002 (2)
C14	0.017 (3)	0.019 (3)	0.018 (3)	0.007 (2)	−0.002 (2)	−0.001 (2)
C15	0.015 (3)	0.027 (3)	0.014 (3)	0.006 (2)	−0.003 (2)	−0.005 (2)
C16	0.031 (3)	0.034 (3)	0.020 (3)	0.003 (3)	0.005 (2)	0.000 (2)
C17	0.034 (3)	0.027 (3)	0.018 (3)	0.011 (3)	−0.001 (2)	0.002 (2)
C18	0.017 (3)	0.028 (3)	0.018 (3)	0.013 (2)	0.000 (2)	0.002 (2)
C19	0.020 (3)	0.022 (3)	0.019 (3)	0.009 (2)	−0.003 (2)	−0.002 (2)
C20	0.017 (3)	0.023 (3)	0.014 (3)	0.013 (2)	0.003 (2)	0.000 (2)
C21	0.019 (3)	0.025 (3)	0.024 (3)	0.011 (3)	0.001 (2)	0.004 (2)
C22	0.022 (3)	0.025 (3)	0.017 (3)	0.012 (2)	0.003 (2)	0.004 (2)
C23	0.021 (3)	0.026 (3)	0.021 (3)	0.005 (2)	0.005 (2)	0.000 (2)
C24	0.028 (3)	0.031 (3)	0.013 (3)	0.010 (3)	0.003 (2)	0.005 (2)
C25	0.019 (3)	0.042 (3)	0.015 (3)	0.015 (3)	0.006 (2)	−0.002 (2)
C26	0.015 (3)	0.022 (3)	0.030 (3)	0.010 (2)	0.004 (2)	0.000 (2)
C27	0.013 (3)	0.025 (3)	0.022 (3)	0.010 (2)	0.003 (2)	0.002 (2)
C28	0.009 (3)	0.026 (3)	0.025 (3)	0.008 (2)	0.000 (2)	0.000 (2)
C29	0.014 (3)	0.020 (3)	0.026 (3)	0.010 (2)	−0.001 (2)	−0.002 (2)
C30	0.020 (3)	0.025 (3)	0.027 (3)	0.012 (2)	0.007 (2)	0.007 (2)
C31	0.027 (3)	0.022 (3)	0.022 (3)	0.012 (2)	0.008 (2)	0.010 (2)
C32	0.030 (3)	0.056 (4)	0.013 (3)	0.017 (3)	0.008 (2)	0.005 (3)

### Geometric parameters (Å, °)

**Table:**

O1—C5	1.235 (6)	C14—C15	1.377 (7)
O2—C12	1.243 (6)	C14—H14	0.9500
O3—C10	1.351 (6)	C15—H15	0.9500
O3—H3	0.8400	C16—H16A	0.9800
O4—C9	1.360 (6)	C16—H16B	0.9800
O4—C16	1.426 (6)	C16—H16C	0.9800
O5—C21	1.242 (6)	C17—C18	1.502 (7)
O6—C28	1.251 (6)	C17—H17A	0.9800
O7—C26	1.356 (6)	C17—H17B	0.9800
O7—H7	0.8400	C17—H17C	0.9800
O8—C25	1.359 (6)	C18—C19	1.390 (7)
O8—C32	1.429 (6)	C18—C31	1.396 (7)
C1—C2	1.509 (7)	C19—C20	1.382 (7)
C1—H1A	0.9800	C19—H19	0.9500
C1—H1B	0.9800	C20—C29	1.429 (7)
C1—H1C	0.9800	C20—C21	1.469 (7)
C2—C3	1.383 (7)	C21—C22	1.470 (7)
C2—C15	1.404 (7)	C22—C23	1.375 (7)
C3—C4	1.394 (7)	C22—C27	1.434 (7)
C3—H3A	0.9500	C23—C24	1.392 (7)
C4—C13	1.402 (7)	C23—H23	0.9500
C4—C5	1.483 (7)	C24—C25	1.376 (8)
C5—C6	1.478 (7)	C24—H24	0.9500
C6—C7	1.372 (7)	C25—C26	1.402 (7)
C6—C11	1.428 (7)	C26—C27	1.390 (7)
C7—C8	1.397 (7)	C27—C28	1.443 (7)
C7—H7A	0.9500	C28—C29	1.476 (7)
C8—C9	1.395 (7)	C29—C30	1.377 (7)
C8—H8	0.9500	C30—C31	1.392 (7)
C9—C10	1.397 (7)	C30—H30	0.9500
C10—C11	1.406 (7)	C31—H31	0.9500
C11—C12	1.459 (7)	C32—H32A	0.9800
C12—C13	1.487 (7)	C32—H32B	0.9800
C13—C14	1.386 (7)	C32—H32C	0.9800
			
C10—O3—H3	109.5	H16A—C16—H16C	109.5
C9—O4—C16	117.9 (4)	H16B—C16—H16C	109.5
C26—O7—H7	109.5	C18—C17—H17A	109.5
C25—O8—C32	117.7 (4)	C18—C17—H17B	109.5
C2—C1—H1A	109.5	H17A—C17—H17B	109.5
C2—C1—H1B	109.5	C18—C17—H17C	109.5
H1A—C1—H1B	109.5	H17A—C17—H17C	109.5
C2—C1—H1C	109.5	H17B—C17—H17C	109.5
H1A—C1—H1C	109.5	C19—C18—C31	118.3 (5)
H1B—C1—H1C	109.5	C19—C18—C17	122.3 (5)
C3—C2—C15	118.9 (5)	C31—C18—C17	119.3 (5)
C3—C2—C1	121.5 (5)	C20—C19—C18	122.1 (5)
C15—C2—C1	119.7 (5)	C20—C19—H19	119.0
C2—C3—C4	121.4 (5)	C18—C19—H19	119.0
C2—C3—H3A	119.3	C19—C20—C29	119.1 (5)
C4—C3—H3A	119.3	C19—C20—C21	120.8 (5)
C3—C4—C13	118.9 (5)	C29—C20—C21	120.0 (4)
C3—C4—C5	119.9 (4)	O5—C21—C22	120.6 (5)
C13—C4—C5	121.1 (4)	O5—C21—C20	120.1 (5)
O1—C5—C6	120.5 (4)	C22—C21—C20	119.2 (5)
O1—C5—C4	121.3 (4)	C23—C22—C27	118.8 (5)
C6—C5—C4	118.2 (4)	C23—C22—C21	121.1 (5)
C7—C6—C11	118.8 (4)	C27—C22—C21	120.1 (4)
C7—C6—C5	121.2 (4)	C22—C23—C24	121.7 (5)
C11—C6—C5	120.0 (4)	C22—C23—H23	119.1
C6—C7—C8	122.5 (4)	C24—C23—H23	119.1
C6—C7—H7A	118.8	C25—C24—C23	120.4 (5)
C8—C7—H7A	118.8	C25—C24—H24	119.8
C9—C8—C7	119.0 (5)	C23—C24—H24	119.8
C9—C8—H8	120.5	O8—C25—C24	125.8 (5)
C7—C8—H8	120.5	O8—C25—C26	115.3 (5)
O4—C9—C10	115.4 (4)	C24—C25—C26	119.0 (5)
O4—C9—C8	124.5 (5)	O7—C26—C27	121.6 (5)
C10—C9—C8	120.1 (4)	O7—C26—C25	116.7 (5)
O3—C10—C9	117.9 (4)	C27—C26—C25	121.7 (5)
O3—C10—C11	121.6 (4)	C26—C27—C22	118.5 (5)
C9—C10—C11	120.5 (4)	C26—C27—C28	120.8 (5)
C10—C11—C6	119.1 (4)	C22—C27—C28	120.7 (5)
C10—C11—C12	119.6 (4)	O6—C28—C27	121.4 (5)
C6—C11—C12	121.3 (4)	O6—C28—C29	119.1 (5)
O2—C12—C11	121.5 (4)	C27—C28—C29	119.5 (5)
O2—C12—C13	120.0 (4)	C30—C29—C20	118.6 (5)
C11—C12—C13	118.5 (4)	C30—C29—C28	121.0 (5)
C14—C13—C4	119.9 (4)	C20—C29—C28	120.3 (5)
C14—C13—C12	119.5 (4)	C29—C30—C31	121.3 (5)
C4—C13—C12	120.6 (4)	C29—C30—H30	119.3
C15—C14—C13	120.6 (5)	C31—C30—H30	119.3
C15—C14—H14	119.7	C30—C31—C18	120.5 (5)
C13—C14—H14	119.7	C30—C31—H31	119.7
C14—C15—C2	120.3 (5)	C18—C31—H31	119.7
C14—C15—H15	119.8	O8—C32—H32A	109.5
C2—C15—H15	119.8	O8—C32—H32B	109.5
O4—C16—H16A	109.5	H32A—C32—H32B	109.5
O4—C16—H16B	109.5	O8—C32—H32C	109.5
H16A—C16—H16B	109.5	H32A—C32—H32C	109.5
O4—C16—H16C	109.5	H32B—C32—H32C	109.5
			
C15—C2—C3—C4	−1.8 (7)	C31—C18—C19—C20	−0.9 (7)
C1—C2—C3—C4	177.5 (4)	C17—C18—C19—C20	−178.7 (5)
C2—C3—C4—C13	1.3 (7)	C18—C19—C20—C29	0.0 (7)
C2—C3—C4—C5	178.1 (4)	C18—C19—C20—C21	179.4 (4)
C3—C4—C5—O1	−1.6 (7)	C19—C20—C21—O5	−0.3 (7)
C13—C4—C5—O1	175.1 (5)	C29—C20—C21—O5	179.1 (5)
C3—C4—C5—C6	176.7 (4)	C19—C20—C21—C22	179.6 (4)
C13—C4—C5—C6	−6.6 (6)	C29—C20—C21—C22	−1.0 (7)
O1—C5—C6—C7	1.9 (7)	O5—C21—C22—C23	−0.8 (8)
C4—C5—C6—C7	−176.5 (4)	C20—C21—C22—C23	179.3 (4)
O1—C5—C6—C11	−176.2 (4)	O5—C21—C22—C27	−179.5 (5)
C4—C5—C6—C11	5.4 (6)	C20—C21—C22—C27	0.6 (7)
C11—C6—C7—C8	−1.7 (7)	C27—C22—C23—C24	−1.1 (7)
C5—C6—C7—C8	−179.8 (4)	C21—C22—C23—C24	−179.8 (5)
C6—C7—C8—C9	1.3 (7)	C22—C23—C24—C25	0.7 (8)
C16—O4—C9—C10	−175.1 (4)	C32—O8—C25—C24	−1.8 (7)
C16—O4—C9—C8	5.6 (7)	C32—O8—C25—C26	178.9 (4)
C7—C8—C9—O4	178.8 (4)	C23—C24—C25—O8	−179.5 (5)
C7—C8—C9—C10	−0.4 (7)	C23—C24—C25—C26	−0.2 (7)
O4—C9—C10—O3	0.8 (6)	O8—C25—C26—O7	−2.4 (7)
C8—C9—C10—O3	−179.9 (4)	C24—C25—C26—O7	178.3 (4)
O4—C9—C10—C11	−179.4 (4)	O8—C25—C26—C27	179.5 (4)
C8—C9—C10—C11	−0.1 (7)	C24—C25—C26—C27	0.2 (7)
O3—C10—C11—C6	179.5 (4)	O7—C26—C27—C22	−178.6 (4)
C9—C10—C11—C6	−0.2 (7)	C25—C26—C27—C22	−0.5 (7)
O3—C10—C11—C12	0.1 (7)	O7—C26—C27—C28	1.7 (7)
C9—C10—C11—C12	−179.6 (4)	C25—C26—C27—C28	179.7 (4)
C7—C6—C11—C10	1.1 (7)	C23—C22—C27—C26	1.0 (7)
C5—C6—C11—C10	179.2 (4)	C21—C22—C27—C26	179.7 (4)
C7—C6—C11—C12	−179.6 (4)	C23—C22—C27—C28	−179.2 (4)
C5—C6—C11—C12	−1.4 (7)	C21—C22—C27—C28	−0.5 (7)
C10—C11—C12—O2	−2.0 (7)	C26—C27—C28—O6	1.1 (7)
C6—C11—C12—O2	178.7 (4)	C22—C27—C28—O6	−178.6 (5)
C10—C11—C12—C13	177.7 (4)	C26—C27—C28—C29	−179.5 (4)
C6—C11—C12—C13	−1.7 (7)	C22—C27—C28—C29	0.8 (7)
C3—C4—C13—C14	0.0 (7)	C19—C20—C29—C30	1.1 (7)
C5—C4—C13—C14	−176.7 (4)	C21—C20—C29—C30	−178.3 (4)
C3—C4—C13—C12	−179.6 (4)	C19—C20—C29—C28	−179.3 (4)
C5—C4—C13—C12	3.6 (7)	C21—C20—C29—C28	1.3 (7)
O2—C12—C13—C14	0.6 (7)	O6—C28—C29—C30	−2.1 (7)
C11—C12—C13—C14	−179.1 (4)	C27—C28—C29—C30	178.4 (4)
O2—C12—C13—C4	−179.7 (4)	O6—C28—C29—C20	178.3 (4)
C11—C12—C13—C4	0.6 (6)	C27—C28—C29—C20	−1.2 (7)
C4—C13—C14—C15	−0.8 (7)	C20—C29—C30—C31	−1.3 (7)
C12—C13—C14—C15	178.9 (4)	C28—C29—C30—C31	179.1 (4)
C13—C14—C15—C2	0.2 (7)	C29—C30—C31—C18	0.4 (7)
C3—C2—C15—C14	1.1 (7)	C19—C18—C31—C30	0.7 (7)
C1—C2—C15—C14	−178.3 (4)	C17—C18—C31—C30	178.6 (5)

### Hydrogen-bond geometry (Å, °)

**Table:**

*D*—H···*A*	*D*—H	H···*A*	*D*···*A*	*D*—H···*A*
O3—H3···O2	0.84	1.80	2.538 (4)	147
O7—H7···O6	0.84	1.81	2.551 (5)	146
